# The synergistic effect of the atherogenic index of plasma and hyperuricemia on the prediction of coronary chronic total occlusion lesion: an observational cross-sectional study

**DOI:** 10.3389/fcvm.2024.1437096

**Published:** 2024-07-23

**Authors:** HongYa Han, XiaoLi Liu, Qi Zhao, ZhiJian Wang, Yan Sun, Xiaoteng Ma, MengChen Li, HanYing Ma, YuYang Liu, DongMei Shi, YuJing Cheng, YuJie Zhou

**Affiliations:** ^1^ Department of Cardiology, Beijing Anzhen Hospital, Beijing Key Laboratory of Precision Medicine of Coronary Atherosclerotic Disease, Clinical Center for Coronary Heart Disease, Capital Medical University, Beijing Institute of Heart Lung and Blood Vessel Disease, Capital Medical University, Beijing, China; ^2^ Department of Cardiology, Cardiovascular Center, Beijing Friendship Hospital, Capital Medical University, Beijing, China; ^3^ Department of General Medicine, Beijing Anzhen Hospital, Capital Medical University, Beijing, China

**Keywords:** coronary artery disease, chronic total occlusion, atherogenic index of plasma, hyperuricemia, prediction

## Abstract

**Background:**

The atherogenic index of plasma (AIP) and hyperuricemia (HUA) have been shown to be closely associated with morbidity and mortality of coronary artery disease. However, studies targeting predictive value of AIP and HUA for chronic total occlusion (CTO) lesions are still lacking.

**Methods:**

In total, 5,238 patients meeting the eligibility criteria were recruited in this analysis. CTO was defined as the condition of lesions without forward blood flow and with over three months of occlusion time. AIP was calculated as log10 [triglycerides (mmol/L)/high-density lipoprotein cholesterol (mmol/L)]. HUA was defined based on sex-specific criteria: serum uric acid 420 and 360 μmol/L for males and females, respectively.

**Results:**

CTO lesions were presented in 907 (17.3%) patients. Compared with patients showing lower AIP levels and non-HUA, the CTO lesion risks increased by 5.225 and 2.765 times in patients with higher AIP levels and HUA. Patients with AIP >0.15 and HUA exhibited the greatest CTO incidence (odds ratio 11.491; 95% confidence interval 9.019–14.641, *P* < 0.001). In addition, AIP combined with HUA had significantly increased effects (a 38.5% increase in CTO risk) relative to the sum of respective effects.

**Conclusion:**

Patients having higher AIP levels and HUA exhibited the highest CTO incidence, in comparison with patients who have the increased single index. AIP combined with HUA displayed significant synergistic effect on the prediction of CTO lesion.

## 1 Introduction

Chronic total occlusion (CTO) is a serious lesion-type of coronary artery disease (CAD), referring to thrombolysis in myocardial infarction (TIMI) 0 flow and lasting at least 3 months (1). It has been shown that CTO occurs in approximately16%–20% of patients with CAD who undergo coronary angiography (CAG) (2, 3). Studies have indicated that percutaneous coronary intervention (PCI) for CTO is characterized by lower success rate, higher complication incidence, longer procedural duration, higher cost, and uncertain clinical benefit (4, 5). As a result, it is vital to identify the CTO-related risk factors and formulate targeted intervention measures for delaying the progression of coronary lesion to CTO, which can thus improve cardiovascular prognosis.

The atherogenic index of plasma (AIP), which is obtained based on lipid profiles including triglycerides (TG) and high-density lipoprotein cholesterol (HDL-C), has been considered to be the alternative and simple marker for plasma atherogenicity; this may be superior to the standard atherosclerotic lipid profiles (6–8). AIP has been indicated to be significantly related to the development, progression, and prognosis of CAD (9–13). The homeostasis of uric acid (UA) depends on its production, excretion, and reabsorption, and disruption of either process may cause hyperuricemia (HUA) (14). Recently, many studies have revealed that HUA is closely associated with the morbidity and mortality of CAD (15–18).

However, currently, studies targeting the predictive value of AIP and HUA for CTO lesions are relatively few. In addition, no previous research is been performed to explore how AIP combined with HUA predicts CTO lesions in patients with CAD patients. Therefore, this study was performed to explore the significance of AIP and HUA for predicting CTO lesions in patients with CAD, as well as further evaluating their synergistic effect on predictive performance.

## 2 Methods

### 2.1 Study population

**Figure 1:**
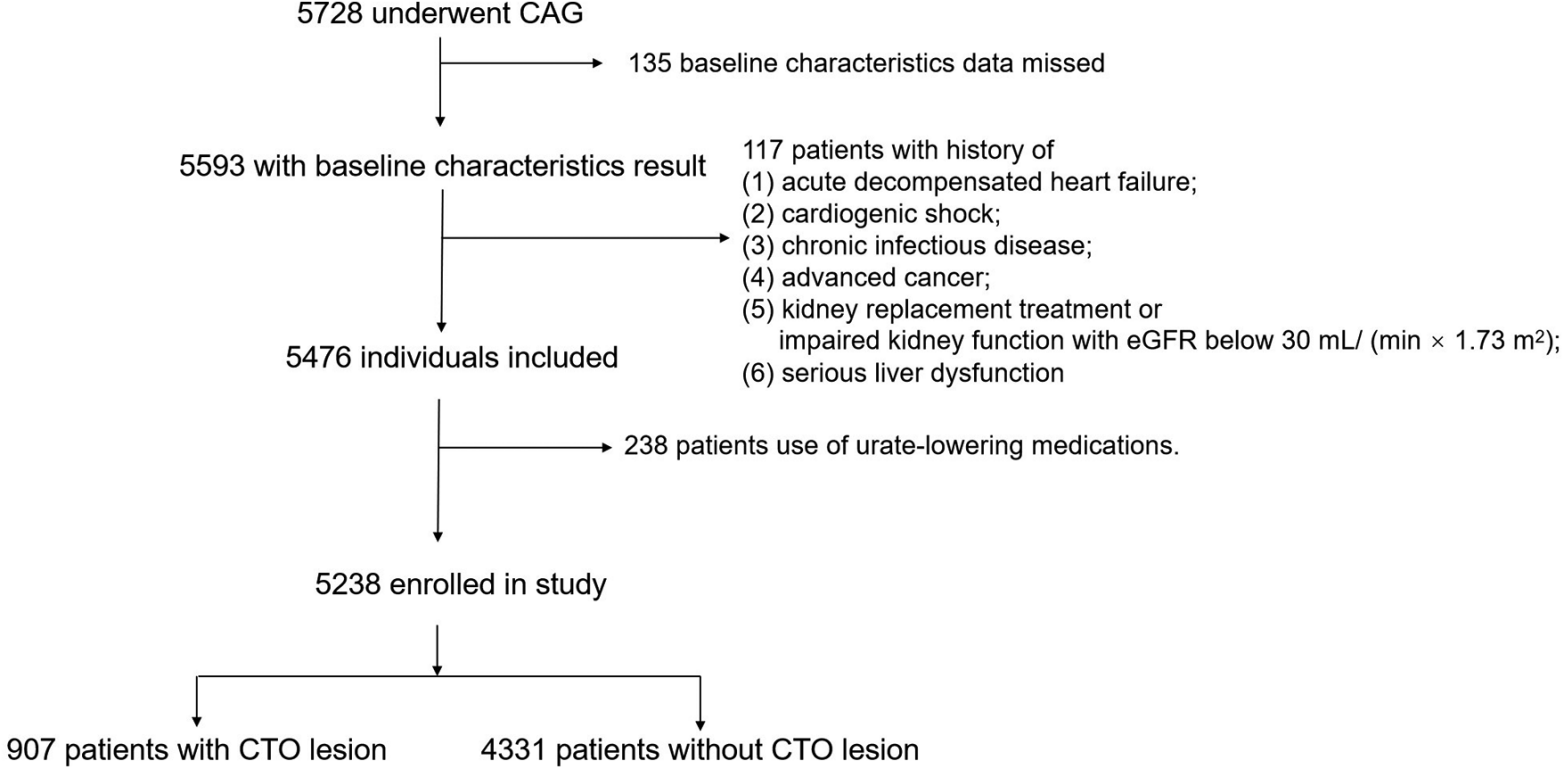
Flow of study participants.

The study consecutively enrolled totally 5,728 patients undergoing CAG in Beijing Anzhen Hospital from August 2018 to February 2019, approved by the Ethics Review Committee of the hospital (approval no. 2018027). Informed consent was obtained from all participants. The patients were presented anonymously. This study included the following patients: (1) those diagnosed with CAD; (2) with age ≤75 and ≥18 years; and (3) receiving CAG. The following patients were excluded: (1) those without basic feature data; (2) using urate-lowering medications; (3) with cardiogenic shock, acute decompensated heart failure, advanced cancer, or chronic infectious disorders; (4) with kidney dysfunction [estimated glomerular filtration rate (eGFR) <30 ml/(min 1.73 m^2^)] or undergoing kidney replacement; and (5) with severe liver dysfunction and having aspartate transaminase and/or alanine transaminase ≥5 times the corresponding upper reference limits. Finally, a total of 5,238 patients were enrolled in this study (Figure 1).

### 2.2 Data extraction

After obtaining written informed consent from the patients, all demographic data and clinical information of the patents were obtained based on the hospital database. CTO lesions were judged by experienced cardiologists, with TIMI 0 forward blood flow of the occluded vessel segment, and with the occlusion lasting for over 3 months. Patients developing two to three-vessel disease were deemed to have multi-vessel disease, while patients developing ≥50% stenosis within the left main coronary artery were considered to have left main disease. The definition of hypertension was systolic blood pressure ≥140 mmHg and/or diastolic blood pressure ≥90 mmHg, or having antihypertensive medication. Diabetes mellitus can be defined as follows: (1) casual blood glucose ≥11.1 mmol/L, or (2) positive result of oral glucose tolerance test, or (3) fasting blood glucose ≥7.0 mmol/L. Smoking was deemed as cumulative (≥6 months) or continuous smoking during the lifetime, while hyperlipidemia as fasting total cholesterol (TC) > 5.17 mmol/L, TG > 1.69 mmol/L, HDL-C < 1.03 mmol/L, low-density lipoprotein cholesterol (LDL-C) > 3.36 mmol/L, and/or having lipid-lowering medication. HUA in adults was based on sex-specific criteria: serum uric acid ≥420 and 360 μmol/L for males and females, respectively. AIP was calculated according to TG and HDL-C: Log10 [TG (mmol/L)/HDL-C (mmol/L)].

### 2.3 Statistical analysis

The median of AIP (0.15) was taken as the optimum threshold based on AIP distribution. Basic patient data were depicted and analyzed following the optimum AIP threshold and diagnosis of HUA. Normally distributed continuous data were indicated by mean ± standard deviation (SD) and analyzed through the Student's *t*-test, as appropriate. Categorical data were indicated by number (percentage) and explored through chi-square tests.

The logistics regression was used to analyze the association of variables with CTO lesion. Three logistics regression models were constructed to evaluate the correlation between AIP/HUA and CTO lesion. Model 1 was adjusted for male gender and age. Variables of *P* < 0.05 upon univariate regression were incorporated into Model 2. Model 3 (the completely adjusted model) incorporated variables showing the univariate relation with adverse events or possible relation to clinical outcome, which included variables of Model 2 as well as age, smoking, and hypertension. To limit the impact of extreme observations, we standardized two interested variables to z score, a score indicating effect size of every unit SD increase. In addition, these two variables were incorporated into models as categorical variables according to median AIP (0.15) and sex-specific HUA criteria.

To evaluate the combined effect of AIP with HUA on predicting CTO lesion, patients were divided into four groups: AIP ≤ 0.15 and non-HUA (group 1), AIP > 0.15 and non-HUA (group 2), AIP ≤ 0.15 and HUA (group 3), or AIP > 0.15 and HUA (group 4). The completely adjusted logistic regression model was used to examine the combined effect on prediction. To explore the association of AIP with HUA, relative excess risk owing to interaction (RERI), synergy index (SI), and attributable proportion (AP) were determined. C-statistics, integrated discrimination improvement (IDI), and net reclassification improvement (NRI) were adopted for analyzing the synergistic prediction effect of AIP and HUA.

## 3 Results

### 3.1 Main patient characteristics

Table 1 displays basic patient characteristics that are classified according to median AIP. In total, this study enrolled totally 5,238 patients, among them, 75.4% were men (*n* = 3,947). Patients with higher AIP were younger and smoking, mostly men, and presented greater body mass index (BMI), heart rate, platelet, hemoglobin (HGB), glycosylated hemoglobin A1c (HbA1c), TC, LDL-C, and UA levels, but lower TG, HDL-C, and eGFR levels. There were more individuals who had acute coronary syndrome (ACS), multi-vessel disease, previous myocardial infarction (MI), PCI, diabetes mellitus, hyperlipidemia, hypertension, and received β-blocker and hypoglycemic therapy.

**Table 1:** Basic patient features based on AIP and UA level.

Variables	All	AIP ≤ 0.15 (*N* = 2,681)	AIP > 0.15 (*N* = 2,557)	*P*-value	Non-hyperuricemia (*N* = 3,960)	Hyperuricemia (*N* = 1,278)	*P*-value
General conditions							
Age (years)	59.72 ± 10.00	61.44 ± 9.64	57.91 ± 10.06	0.019	60.11 ± 9.53	58.48 ± 11.27	<0.001
Male, *n* (%)	3,947 (75.4)	1,969 (73.4)	1,978 (77.4)	<0.001	2,996 (75.7)	951 (74.4)	0.371
BMI (kg/m^2^)	26.05 ± 3.18	25.58 ± 3.20	26.54 ± 3.10	<0.001	25.86 ± 3.11	26.63 ± 3.33	<0.001
Heart rate (bpm)	72.44 ± 10.66	72.03 ± 10.42	72.86 ± 10.90	0.005	72.41 ± 10.44	72.52 ± 11.33	0.749
LVEF (%)	61.33 ± 8.02	61.64 ± 7.79	61.01 ± 8.24	0.004	61.53 ± 7.66	60.73 ± 9.02	0.002
Previous MI, *n* (%)	701 (13.4)	332 (12.4)	369 (14.4)	0.031	202 (15.8)	499 (12.6)	0.004
Previous stroke, *n* (%)	273 (5.2)	140 (5.2)	133 (5.2)	1.000	77 (6.0)	196 (4.9)	0.147
Previous PCI, *n* (%)	1,292 (24.7)	615 (22.9)	677 (26.5)	0.003	309 (24.2)	983 (24.8)	0.654
Previous CABG, *n* (%)	129 (2.5)	62 (2.3)	67 (2.6)	0.477	35 (2.7)	94 (2.4)	0.468
Left main disease, *n* (%)	500 (9.5)	277 (10.3)	223 (8.7)	0.048	122 (9.5)	378 (9.5)	1.000
Multi-vessel disease, *n* (%)	2,067 (39.5)	1,019 (38.0)	1.48 (41)	0.029	499 (39.0)	1,568 (39.6)	0.742
CTO (%)	907 (17.3)	183 (6.8)	724 (28.3)	<0.001	516 (13.0)	396 (30.6)	<0.001
ISR (%)	206 (3.9)	96 (3.6)	110 (4.3)	0.200	52 (4.1)	154 (3.9)	0.804
Average stent diameter (mm)	2.98 ± 0.44	2.97 ± 0.44	2.99 ± .045	0.06	2.97 ± 0.44	3.00 ± 0.45	0.053
Total stent length (mm)	38.06 ± 24.25	37.88 ± 24.41	38.24 ± 24.09	0.593	37.76 ± 24.07	38.99 ± 24.80	0.116
PCI indication							
ACS, *n* (%)	1,000 (19.1)	465 (17.3)	535 (20.9)	<0.001	226 (17.7)	774 (19.5)	0.152
CCS, *n* (%)	4,238 (80.9)	2,216 (82.7)	2,022 (79.1)	<0.001	1,052 (82.3)	3,186 (80.5)	0.152
Risk factors, *n* (%)							
Smoking	2,730 (52.1)	1,310 (48.9)	1,420 (55.5)	<0.001	679 (53.1)	2,051 (51.8)	0.421
Diabetes mellitus	1,884 (36.0)	881 (32.9)	1,003 (39.2)	<0.001	411 (32.2)	1,473 (37.2)	0.001
Hypertension	3,454 (65.9)	1,699 (63.4)	1,755 (68.6)	<0.001	936 (73.2)	2,518 (63.6)	<0.001
Hyperlipidemia	3,906 (74.6)	1,963 (73.2)	1,943 (76.0)	0.022	945 (73.9)	2,961 (74.8)	0.555
Laboratory measurements							
PLT (10^9^ /L)	224.51 ± 60.13	221.93 ± 59.56	227.21 ± 60.62	0.001	223.75 ± 59.79	226.84 ± 61.15	0.110
HGB (g/L)	141.25 ± 16.38	140.48 ± 15.78	142.06 ± 16.96	<0.001	141.62 ± 15.85	140.11 ± 17.87	0.004
HbA1c (%)	6.59 ± 1.41	6.48 ± 1.33	6.70 ± 1.47	<0.001	6.63 ± 1.43	6.47 ± 1.31	<0.001
TC (mmol/L)	4.15 ± 1.04	4.01 ± 0.97	4.30 ± 1.09	<0.001	4.13 ± 1.03	4.22 ± 1.05	0.006
TG (mmol/L)	1.19 ± 0.48	1.26 ± 0.46	1.14 ± 0.50	<0.001	1.20 ± 0.50	1.20 ± 0.44	0.984
HDL-C (mmol/L)	1.11 ± 0.29	1.20 ± 0.28	1.01 ± 0.27	<0.001	1.11 ± 0.29	1.10 ± 0.29	0.074
LDL-C (mmol/L)	2.45 ± 0.86	2.40 ± 0.83	2.52 ± 0.88	<0.001	2.44 ± 0.85	2.49 ± 0.88	0.079
eGFR (ml/min/1.73 m^2^)	96.07 ± 18.45	97.23 ± 17.50	94.86 ± 19.32	<0.001	98.63 ± 16.70	88.15 ± 21.17	<0.001
UA (μmol/L)	353.42 ± 88.99	336.16 ± 81.53	371.52 ± 92.83	<0.001	316.51 ± 58.18	467.82 ± 68.28	<0.001
AIP	0.16 ± 0.28	−0.049 ± 0.16	0.37 ± 0.20	0.001	0.13 ± 0.27	0.25 ± 0.28	<0.001
Medication at the time of admission, *n* (%)							
β-blocker (%)	3,182 (60.7)	1,550 (57.8)	1,632 (63.8)	<0.001	791 (61.9)	2,391 (60.4)	0.340
Statin (%)	5,175 (98.8)	2,651 (98.9)	2,524 (98.7)	0.613	1,264 (98.9)	3,911 (98.8)	0.769
Aspirin (%)	5,150 (98.3)	2,636 (98.3)	2,514 (98.3)	1.000	1,247 (97.6)	3,903 (98.6)	0.023
ADP inhibitor (%)	3,837 (73.3)	1,944 (72.5)	1,893 (74.0)	0.223	877 (68.6)	2,960 (74.7)	<0.001
Hypoglycemic drugs (%)	1,502 (28.7)	499 (18.6)	1,003 (39.2)	<0.001	322 (25.2)	1,180 (29.8)	0.002

Data are indicated by mean + SD, or frequency *n* (percent).

BMI, body mass index; LVEF, left ventricle ejection fraction; MI, myocardial Infarction; PCI, percutaneous coronary intervention; CABG, coronary artery bypass grafting; ISR, in-stent restenosis; CCS, chronic coronary syndrome; PLT, platelet.

Patients were classified as HUA or non-HUA group (Table 1). Compared with the non-HUA subjects, HUA patients were younger, and presented greater BMI, TC, and AIP levels, with lower left ventricle ejection fraction (LVEF), HGB, HbA1c, and eGFR levels. In addition, there were more individuals who had previous MI, and diabetes mellitus, and were treated with aspirin, adenosine diphosphate (ADP) inhibitor, and hypoglycemic therapy.

### 3.2 Respective predictive value of AIP and HUA for CTO

In our study, CTO lesions were presented in 907 (17.3%) patients. The rate of CTO lesion was significantly higher in patients with higher AIP (*P* < 0.001). Patients with HUA had a significantly increased risk of CTO (*P* < 0.001) compared to those without HUA. Univariate logistics regression analysis showed that AIP and HUA were significantly associated with CTO lesion (Table 2). The predictive value of AIP and HUA remained significant after modifying for other cardiovascular risk factors (Table 3). Compared to patients with low levels of AIP and non-HUA, the risk of CTO lesion was 5.225 times higher for individuals with a high AIP and 2.765 times higher for individuals with HUA.

**Table 2:** Univariate logistics regression for CTO.

Variables	OR	95% CI	*P*-value
Age	0.996	0.989–1.003	0.248
Male	1.178	1.002–1.385	0.047
BMI	1.019	0.996–1.042	0.101
Heart rate (bpm)	1.004	0.997–1.011	0.231
LVEF	0.993	0.985–1.002	0.127
Previous MI	1.163	0.949–1.425	0.144
Previous stroke	0.887	0.636–1.239	0.483
Previous PCI	0.946	0.800–1.118	0.513
Previous CABG	1.809	1.219–2.685	0.003
Left main disease	0.614	0.463–0.813	0.001
Multi-vessel disease	1.064	0.919–1.231	0.408
ISR	1.352	0.964–1.897	0.081
Average stent diameter	0.907	0.770–1.069	0.243
Total stent length	1.002	0.999–1.005	0.199
ACS	0.781	0.644–0.947	0.012
Smoking	0.996	0.863–1.150	0.958
Diabetes mellitus	0.812	0.697–0.946	0.007
Hypertension	1.061	0.911–1.235	0.445
Hyperlipidemia	1.056	0.894–1.246	0.521
PLT	1.001	1.000–1.002	0.226
HGB	1.000	0.996–1.005	0.862
HbA1c	0.969	0.920–1.021	0.240
TC	1.448	1.356–1.547	<0.001
LDL-C	1.337	1.236–1.447	<0.001
eGFR	0.991	0.987–0.994	<0.001
β-blocker	0.967	0.836–1.119	0.654
Statin	1.445	0.686–3.045	0.333
Aspirin	0.707	0.427–1.171	0.178
ADP inhibitor	1.154	0.978–1.362	0.090
Hypoglycemic drugs	1.151	0.985–1.344	0.077

CABG, coronary artery bypass grafting; ISR, in-stent restenosis; PLT, platelet.

**Table 3:** Multivariate logistics regression for CTO.

Variables	OR (95% CI)		
	Model 1	Model 2	Model 3
AIP			
Per 1 × 10^−2^ unit increase	1.014 (1.011–1.017)*	1.011 (1.008–1.013)*	1.010 (1.007–1.013)*
Per SD increase	1.476 (1.373–1.587)*	1.342 (1.240–1.452)*	1.331 (1.227–1.444)*
AIP ≤ 0.15	Reference		
AIP > 0.15	5.6 (4.698–6.675)*	5.122 (4.288–6.118)*	5.225 (4.362–6.259)*
UA			
Per unit increase	1.005 (1.004–1.006)*	1.005 (1.004–1.005)*	1.005 (1.004–1.005)*
Per SD increase	1.559 (1.450–1.675)*	1.501 (1.388–1.622)*	1.497 (1.382–1.621)*
Non-HUA	Reference		
HUA	2.927 (2.516–3.404)*	2.781 (2.370–3.263)*	2.765 (2.350–3.254)*

Model 1: Adjusted for age and male gender. Model 2: Adjusted for male gender, Previous CABG, Left main disease, ACS, Diabetes mellitus, TC, LDL-C, and eGFR. Model 3: Adjusted for male gender, Previous CABG, Left main disease, ACS, Diabetes mellitus, TC, LDL-C, eGFR, age, smoking, and hypertension.

* *P* < 0.001.

### 3.3 Corresponding prediction effect of AIP and HUA on CTO

All subjects were classified into one of four groups following median AIP and sex-specific HUA criteria. Logistics regression was performed to compare CTO lesion risk of the four groups (Table 4). Therefore, the greatest CTO lesion risk was determined in those having AIP > 0.15 and HUA, while the cumulative CTO lesion rate significantly decreased among patients having AIP ≤ 0.15 and without HUA (*P* < 0.001). The pooled significance of AIP and HUA for predicting CTO was confirmed through multivariate logistics regression. CTO risk was the highest in those having AIP > 0.15 and HUA [odds ratio (OR) = 11.491; 95% confidence interval (CI) 9.019–14.641, *P* < 0.001]. The adjusted OR for CTO was 1.938 (95% CI 1.373–2.735) in patients with AIP ≤ 0.15 and non-HUA, relative to patients of Group 1 (Table 4).

**Table 4:** Pooled effect of AIP and HUA on CTO.

	Univariate regression	Multivariate regression
	OR (95% CI)	OR (95% CI)
AIP ≤ 0.15 and non-HUA	Reference	
AIP > 0.15 and non-HUA	1.981 (1.413–2.778)*	1.938 (1.373–2.735)*
AIP ≤ 0.15 and HUA	4.487 (3.638–5.534)*	4.468 (3.600–5.545)*
AIP > 0.15 and HUA	11.458 (9.148–14.351)*	11.491 (9.019–14.641)*

Adjusted for male gender, previous CABG, left main disease, ACS, diabetes mellitus, TC, LDL-C, eGFR, age, smoking, and hypertension.

* *P* < 0.001.

### 3.4 Subgroup analysis

**Figure 2:**
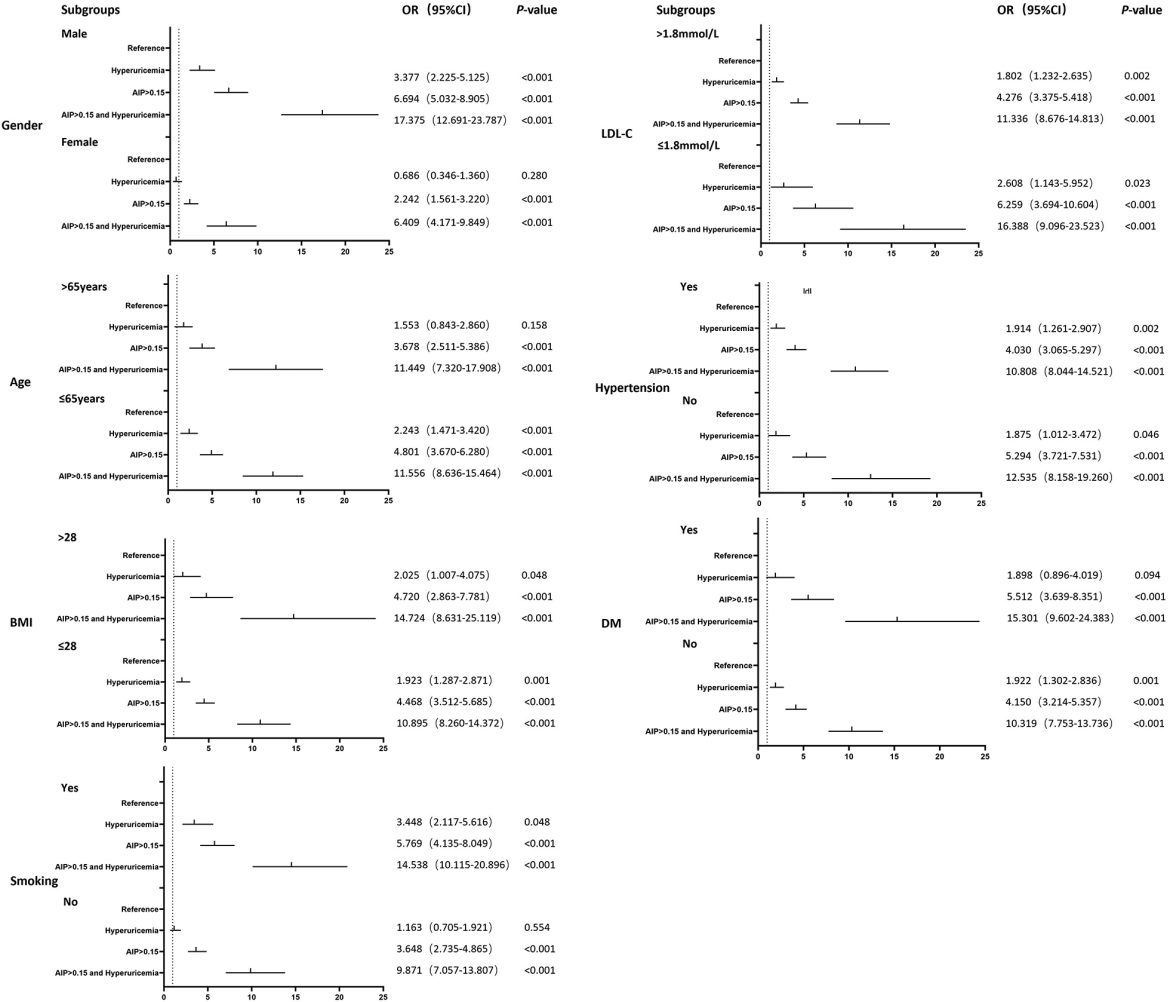
Subgroup analysis.

Subgroup analyses stratified by age, sex, BMI, smoking, hypertension, diabetes mellitus, and hyperlipidemia were performed. Figure 2 presents the pooled relation between AIP/HUA and CTO lesion in different subgroups. In general, patients having higher AIP levels and HUA were significantly associated with CTO lesion, in all subgroups (Figure 2).

### 3.5 Interaction between AIP and HUA

Based on interaction analysis, the pooled effect of AIP with HUA significantly increased relative to the respective single effects, with the CTO risk being increased by 38.5% (Table 5).

**Table 5:** Synergistic relation of AIP with HUA.

	Value	Lower	Upper
RERI	3.423	1.279	5.567
AP	0.385	0.199	0.571
SI	1.766	1.229	2.538

### 3.6 The incremental predictive effect of AIP with HUA

Based on C-statistic, continuous NRI, and IDI, adding AIP or HUA into the baseline model moderately but significantly improved the prediction effect. Afterward, both AIP and HUA were added into the baseline model to maximally improve model prediction. The addition of AIP and HUA markedly enhanced the discrimination and reclassification performance of the baseline model, with the category-free NRI and IDI being 0.411 and 0.027, respectively (*P* < 0.001) (Table 6).

**Table 6:** C-statistic NRI and IDI for prediction significance of diverse models.

	C-statistic (95% CI)	Continuous NRI (95% CI)	IDI (95% CI)
Model 3	0.662 (0.643–0.682)*	Reference	Reference
Model 3 + AIP	0.680 (0.661–0.699)*	0.354 (0.283–0.424)*	0.009 (0.006–0.01)*
Model 3 + HUA	0.689 (0.670–0.708)*	0.303 (0.232–0.374)*	0.021 (0.016–0.026)*
Model 3 + AIP + HUA	0.693 (0.674–0.712)*	0.411 (0.342–0.479)*	0.027 (0.021–0.033)*

* *P* < 0.001.

### 3.7 The novel cut-off vs. classic cut-off predictive value of AIP with HUA

Recently, a novel cut-off value of hyperuricemia has been described that had a better relationship with cardiovascular diseases. The Uric acid Right for heArt Health (URRAH) study, a cross-sectional analysis including 26,971 individuals, had found a cut-off of 5.1 mg/dl for women and 5.6 mg/dl for men (19).

We made further analysis based on these criteria. Patients were also classified into the HUA or non-HUA group (Supplementary Table S1). Compared with non-HUA subjects, patients with HUA were mostly men, younger and smoking, and presented greater BMI, TG, HDL-C, AIP levels and average stent diameter, but lower LVEF, HGB, HbA1c, and eGFR levels. In addition, there were more individuals who had previous MI, diabetes mellitus, and hypertension, and were treated with hypoglycemic therapy.

Compared to patients with non-HUA, the risk of CTO lesion was 1.545 times higher for individuals with HUA (Supplementary Table S2). Patients were classified into one of four groups following median AIP and novel HUA criteria. CTO risk was the highest in those having AIP > 0.15 and HUA (OR = 5.882; 95% CI 4.581–7.552, *P* < 0.001) (Supplementary Table S3). The odds ratio was lower than the classic criteria. In subgroup analyses, patients with higher AIP levels and HUA were also significantly associated with CTO lesion, in all subgroups (Supplementary Figure S1).

Based on interaction analysis, the pooled effect of AIP with HUA increased relative to the respective single effects, with the CTO risk being increased by 34.9% (Supplementary Table S4), which was similar to that of the classic criteria. The category-free NRI and IDI had values of 0.750 and 0.073, respectively (*P* < 0.001), which had better improvement on the prediction effect.

The novel HUA cut-off showed excellent predictive value and is worth further exploration in future studies.

## 4 Discussion

Our major results include the following: (1) Both high AIP and HUA showed significant independent association with CTO lesion. (2) Patients having high AIP levels and HUA exhibited the highest CTO risk in relation to an increased single index, no matter the subgroup. (3) The pooled effect of AIP with HUA significantly increased relative to the sum of the respective single effects. (4) The addition of AIP and HUA to the baseline model maximally improved risk prediction for CTO.

### 4.1 Characteristics and prognosis of CTO lesions

CTO lesions are mainly characterized by complete occlusion (TIMI flow grade 0) and duration greater than 3 months (1). Recently, the CTO lesion recanalization success rate through PCI steadily increased with the growth of operator experience, technological advancement, and technical development. However, the overall success rate of CTO lesion recanalization through PCI is low (20, 21). In addition, it was also shown that CTO-related PCI is associated with higher risk of complications including perforation, side branch occlusion, and myocardial infarction, when compared with PCI performed in non-CTO lesions (21–24). CTO-related PCI often takes a long time to operate on, which inevitably causes higher radiation doses for both the patient and the operator (25). Moreover, since CTO-related PCI procedures require more advanced consumables and auxiliary means (such as intravascular ultrasound, and optical coherence tomography), the cost is inevitably higher (5). More importantly, whether invasive strategies for CTO lesions can improve outcomes in patients with CAD is controversial (26–29). One recent meta-analysis including randomized controlled trials that compared the therapeutic effects of PCI with the optimal medical therapy alone demonstrated that CTO-related PCI did not improve long-time prognosis of patients with CAD (30).

Considering the lower success rate, higher complication incidence, longer procedural duration, higher cost, and uncertain clinical benefit, identifying the risk factors for CTO lesions and formulating targeted interventions as early as possible plays a crucial role in delaying the progression of coronary lesion to CTO and improving the prognosis of patients with CAD.

### 4.2 Associations between AIP, HUA, and CAD

AIP, which is simply calculated from common lipid profiles including TG and HDL-C, is proposed to be the novel risk predictor of the development, progression, and prognosis of CAD. According to a case-control study from Cai et al. (11), it was concluded that AIP is closely related to CAD, indicating that it may be a useful risk predictor for CAD. According to the results from the Indian Atherosclerosis Research Study (31), the addition of family history and AIP into conventional cardiovascular risk factors improved risk discrimination for CAD (C-index: 0.864–0.873). Previous studies indicated a significant relationship of AIP with the progression of atheroma volume and coronary artery calcification determined with multi-detector computed tomography for participants free of cardiovascular disease at baseline (6, 32). Previous studies also revealed that there was a positive correlation between AIP and the synergy between PCI with taxus and cardiac surgery (SYNTAX) score (33). In a prospective cohort study including 2,676 middle-aged adults with a median follow-up of 7.8 years, when conventional risk factors were adjusted, an increase in AIP was a significant predictor of CAD development (10). The study of Qin et al. (13) further investigated the role of AIP in predicting adverse cardiovascular outcomes among patients with CAD, and the results showed that the increase of AIP showed significant relation to the higher incidence of major adverse cardiovascular/cerebrovascular events.

The relationship of HUA with the CAD morbidity and mortality is also well elucidated in previous studies. Niskanen et al. (34) prospectively recruited totally 1,423 middle-aged men initially free of cardiovascular disease and followed them for 11.9 years. The results demonstrated that HUA strongly predicted cardiovascular mortality among normal middle-aged men, regardless of other risk factors. In addition, another large-scale epidemiological study involving participants aged 25–74 years with an average of 16.4 years’ follow-up also indicated that HUA was significantly and independently associated with cardiovascular mortality risk (35). In addition, previous studies also demonstrated that HUA was significantly and independently related to 30-day and long-term mortality after acute myocardial infarction, and closely correlated with Killip class (36). The predictive value of HUA for the morbidity and mortality of CAD has been also verified by various meta-analyses (15–17). Even so, not all the studies on the relationship between HUA and CAD show a significant association. Previous studies found the absence of a role for HUA in determining CAD as well as left ventricular diastolic dysfunction, which indicated that HUA could act on the heart in an early phase of the disease, whereas other factors were involved in the advanced stages (37). This finding suggested that the question of how HUA impacts the progression of disease is worth further investigation.

Currently, studies targeting the predictive value of AIP and HUA for CTO lesions are few. Our study fills in the gaps of previous research, since it first revealed the significant association between AIP and HUA and the presence of CTO lesions. Then, it further provided the important evidence supporting the synergy of AIP with HUA for the prediction of CTO lesion risk.

### 4.3 Potential mechanisms mediating the atherogenic effect of AIP and HUA

AIP is suggested to show a positive relationship with serum malondialdehyde contents that reflect oxidative stress, and the latter is significantly related to coronary atherosclerosis (38). In addition, AIP is closely associated with epicardial adipose tissue (39), which has been shown to directly influence the progression of coronary atherosclerosis (40). AIP has also been demonstrated to exhibit direct and independent association with decreased arterial stiffness (41) and coronary flow reserve (42), both of which are vital risk factors inducing atherosclerosis. In addition, certain studies have indicated that HUA can enhance atherosclerosis development through inflammation, endothelial dysfunction, vascular smooth muscle cell proliferation, oxidative stress, and renin-angiotensin-aldosterone system activation (43–46). The above-mentioned factors may be important potential mechanisms that can mediate the atherogenic effect of AIP and HUA.

Previous studies have indicated that AIP is significantly related to various CAD-related risk factors including obesity, diabetes mellitus, and hypertension (10, 47, 48). The association of HUA with hypertension and metabolic syndrome was also verified by previous studies (49, 50). This may be another potential mechanism that can lead to atherosclerosis progression in response to AIP and HUA.

This study found a significant synergistic effect of AIP and HUA in predicting CTO lesions. The synergistic effect may be explained by insulin resistance. It has also been illustrated that insulin resistance is mainly characterized as hyperglycemia, hyperinsulinemia, central obesity, and dyslipidemia, particularly the elevated fasting TG and reduced HDL-C. Therefore, AIP, which is determined based on TG and HDL-C, indicates the level of insulin resistance to some extent. Insulin resistance can decrease UA excretion while promoting UA reabsorption. In addition, HUA exerts adverse influence on glucose absorption into skeletal muscle, and mediates oxidative alterations of adipocytes, resulting in insulin resistance (43, 44). They reinforce each other mutually, finally contributing to atherosclerosis development.

### 4.4 Study limitations

However, this study still has the following limitations. First, this is a single-center, retrospective observational study. The validation and generalizability of the findings in this cohort should be carried out based on independent validation samples. Second, all the subjects included in this study were Chinese. The model was created only from the patients who underwent coronary angiography and the severity of CTO was not recorded, probably causing selection bias. Third, despite the fact that subjects receiving urate-lowering drugs were excluded, medications including statins and hypoglycemic agents may have potential influences on evaluating AIP and UA. As is well known, diuretics have a significant impact on UA levels. The database in this study does not provide detailed records of diuretic use, which may have effect on the results. Although a previous study has shown that diuretic-related hyperuricemia carries a similar risk of cardiovascular events and all-cause mortality compared with patients with hyperuricemia in the absence of diuretic therapy (51), it is still unclear whether this result is applicable to the study with CTO lesions as the outcome. Additional data are required, and further research is necessary. Finally, dynamic changes from continuous monitoring of AIP and UA may provide more valuable information, but these were not covered in our study.

## 5 Conclusion

To conclude, both higher AIP and HUA are significantly related to CTO lesion. Patients who have higher AIP and HUA exhibit the highest CTO risk in comparison with those having an increased single index. AIP and HUA display a significant synergistic effect on predicting CTO lesions.

## Data Availability

The original contributions presented in the study are included in the article/**Supplementary Material**, further inquiries can be directed to the corresponding authors.

## Supplementary material

Supplementary Figure S1 Subgroup analysis.
