# Supplementary material for: The synergistic effect of the atherogenic index of plasma and hyperuricemia on the prediction of coronary chronic total occlusion lesion: an observational cross-sectional study
Source: Front Cardiovasc Med. 2024 Jul 23;11:1437096. doi: 10.3389/fcvm.2024.1437096 (PMC11300285; doi:10.3389/fcvm.2024.1437096)
Supplement: Supplementary file 1 [file Datasheet1.docx]

## Table S1. Basic patient features based on AIP and UA level.

| Variables | All | AIP≤0.15  (N=2681) | AIP>0.15  (N=2557) | P-value | Non-Hyperuricemia  (N=2171) | Hyperuricemia  (N=3067) | P-value |
| --- | --- | --- | --- | --- | --- | --- | --- |
| General conditions | | | | | | | |
| Age (years) | 59.72±10.00 | 61.44±9.64 | 57.91±10.06 | 0.019 | 60.94±9.20 | 58.85±10.469 | <0.001 |
| Male, n (%) | 3947 (75.4) | 1969 (73.4) | 1978 (77.4) | <0.001 | 1531 (70.5) | 2416 (78.8) | <0.001 |
| BMI (kg/m^2^) | 26.05±3.18 | 25.58±3.20 | 26.54±3.10 | <0.001 | 25.60±3.13 | 26.36±3.19 | <0.001 |
| Heart rate(bpm) | 72.44±10.66 | 72.03±10.42 | 72.86±10.90 | 0.005 | 72.65±10.37 | 72.28±10.87 | 0.217 |
| LVEF (%) | 61.33±8.02 | 61.64±7.79 | 61.01±8.24 | 0.004 | 61.60±7.58 | 61.15±8.31 | 0.047 |
| Previous MI, n (%) | 701 (13.4) | 332 (12.4) | 369 (14.4) | 0.031 | 255 (11.7) | 446 (14.5) | 0.003 |
| Previous stroke, n (%) | 273 (5.2) | 140 (5.2) | 133 (5.2) | 1.000 | 107(4.9) | 166 (5.4) | 0.45 |
| Previous PCI, n (%) | 1292 (24.7) | 615 (22.9) | 677 (26.5) | 0.003 | 762 (24.8) | 530 (24.4) | 0.745 |
| Previous CABG, n (%) | 129 (2.5) | 62 (2.3) | 67 (2.6) | 0.477 | 43 (2.0) | 86 (2.8) | 0.07 |
| Left main disease, n (%) | 500 (9.5) | 277 (10.3) | 223 (8.7) | 0.048 | 212 (9.8) | 288 (9.4) | 0.668 |
| Multivessel disease, n (%) | 2067 (39.5) | 1019 (38.0) | 1.48 (41) | 0.029 | 884 (40.7) | 1183 (38.6) | 0.121 |
| CTO (%) | 907 (17.3) | 183 (6.8) | 724 (28.3) | <0.001 | 284 (13.1) | 623 (20.3) | <0.001 |
| ISR (%) | 206 (3.9) | 96 (3.6) | 110 (4.3) | 0.200 | 79(3.6) | 127 (4.1) | 0.387 |
| Average stent diameter (mm) | 2.98±0.44 | 2.97±0.44 | 2.99±.045 | 0.06 | 2.96±0.43 | 2.99±0.45 | 0.005 |
| Total stent length (mm) | 38.06±24.25 | 37.88±24.41 | 38.24±24.09 | 0.593 | 37.38±23.89 | 38.54±24.50 | 0.087 |
| PCI indication | | | | |  |  |  |
| ACS, n (%) | 1000 (19.1) | 465 (17.3) | 535 (20.9) | <0.001 | 412 (19.0) | 588 (19.2) | 0.887 |
| CCS, n (%) | 4238 (80.9) | 2216 (82.7) | 2022 (79.1) | <0.001 | 1759 (81.0) | 2479 (80.8) | 0.887 |
| Risk factors, n (%) | | | | |  |  |  |
| Smoking | 2730 (52.1) | 1310 (48.9) | 1420 (55.5) | <0.001 | 1066 (49.1) | 1664 (54.3) | <0.001 |
| Diabetes mellitus | 1884 (36.0) | 881 (32.9) | 1003 (39.2) | <0.001 | 894 (41.2) | 990 (32.3) | <0.001 |
| Hypertension | 3454 (65.9) | 1699 (63.4) | 1755 (68.6) | <0.001 | 1367 (63.0) | 2087 (68.0) | <0.001 |
| Hyperlipidemia | 3906 (74.6) | 1963 (73.2) | 1943 (76.0) | 0.022 | 1624 (74.8) | 2282 (74.4) | 0.748 |
| Laboratory measurements | | | | | | | |
| PLT (10^9^ /L) | 224.51±60.13 | 221.93±59.56 | 227.21±60.62 | 0.001 | 223.84±59.90 | 224.98±60.30 | 0.497 |
| HGB (g/L) | 141.25±16.38 | 140.48±15.78 | 142.06±16.96 | <0.001 | 140.20±15.50 | 141.99±16.94 | <0.001 |
| HbA1c (%) | 6.59±1.41 | 6.48±1.33 | 6.70±1.47 | <0.001 | 6.77±1.54 | 6.46±1.29 | <0.001 |
| TC (mmol/L) | 4.15±1.04 | 4.01±0.97 | 4.30±1.09 | <0.001 | 4.13±1.01 | 4.16±1.06 | 0..269 |
| TG (mmol/L) | 1.19±0.48 | 1.26±0.46 | 1.14±0.50 | <0.001 | 1.23±0.58 | 1.78±0.40 | <0.001 |
| HDL-C (mmol/L) | 1.11±0.29 | 1.20±0.28 | 1.01±0.27 | <0.001 | 1.14±0.30 | 1.09±0.28 | <0.001 |
| LDL-C (mmol/L) | 2.45±0.86 | 2.40±0.83 | 2.52±0.88 | <0.001 | 2.44±0.82 | 2.47±0.88 | 0.191 |
| eGFR (mL/min/1.73 m^2^) | 96.07±18.45 | 97.23±17.50 | 94.86±19.32 | <0.001 | 100.20±15.83 | 93.16±19.57 | <0.001 |
| UA (μmol/L) | 353.42±88.99 | 336.16±81.53 | 371.52±92.83 | <0.001 | 275.01±40.79 | 408.93±7.10 | <0.001 |
| AIP | 0.16±0.28 | -0.049±0.16 | 0.37±0.20 | 0.001 | 0.07±0.26 | 0.18±0.27 | <0.001 |
| Medication at the time of admission, n (%) | | | | | | | |
| β-blocker (%) | 3182 (60.7) | 1550 (57.8) | 1632 (63.8) | <0.001 | 1302 (60.0) | 1880(61.3) | 0.343 |
| Statin (%) | 5175 (98.8) | 2651 (98.9) | 2524 (98.7) | 0.613 | 2150 (99.0) | 3025 (98.6) | 0.201 |
| Aspirin (%) | 5150 (98.3) | 2636 (98.3) | 2514 (98.3) | 1.000 | 2137(98.4) | 3013 (98.2) | 0.663 |
| ADP inhibitor (%) | 3837 (73.3) | 1944 (72.5) | 1893 (74.0) | 0.223 | 1607 (74.0) | 2230 (72.7) | 0.296 |
| Hypoglycemic drugs (%) | 1502 (28.7) | 499 (18.6) | 1003 (39.2) | <0.001 | 704 (32.4) | 798 (26.0) | <0.001 |

Data are indicated by mean + SD, or frequency n (percent).

BMI body mass index, LVEF left ventricle ejection fraction, MI myocardial Infarction, PCI percutaneous coronary intervention, CABG coronary artery bypass grafting, CTO chronic total occlusion, ISR in-stent restenosis, ACS acute coronary syndrome, CCS chronic coronary syndrome, PLT platelet, HGB hemoglobin, HbA1c glycosylated hemoglobin A1c, TC total cholesterol, TG triglyceride, HDL-C, high-density lipoprotein-cholesterol, LDL-C, low-density lipoprotein-cholesterol, eGFR estimated glomerular filtration rate, UA uric acid, AIP atherogenic index of plasma, ADP adenosine diphosphate.

## Table S2. Multivariate Logistics regression for CTO.

| Variables | OR (95% CI) | | |
| --- | --- | --- | --- |
|  | Model 1 | Model 2 | Model 3 |
| UA | | | |
| Per unit increase | 1.005 (1.004-1.006) ^*^ | 1.005 (1.004-1.005) ^*^ | 1.005 (1.004-1.005) ^*^ |
| Per SD increase | 1.559 (1.450-1.675) ^*^ | 1.501 (1.388-1.622) ^*^ | 1.497 (1.382-1.621) ^*^ |
| Non-HUA | Reference | | |
| HUA | 1.716 (1.471-2.002) ^*^ | 1.575 (1.342-1.847) ^*^ | 1.545 (1.315-1.816)^*^ |

Model 1: Adjusted for age and male gender.

Model 2: Adjusted for male gender, Previous CABG, Left main disease, ACS, Diabetes mellitus, TC, LDL-C, and eGFR.

Model 3: Adjusted for male gender, Previous CABG, Left main disease, ACS, Diabetes mellitus, TC, LDL-C, eGFR, age, smoking, and hypertension.

*P<0.001

AIP atherogenic index of plasma, UA uric acid, SD standard deviation, HUA hyperuricemia, OR odds ratio, and CI confidence interval.

## Table S3. Pooled effect of AIP and HUA on CTO.

|  | Univariate regression | Multivariate regression |
| --- | --- | --- |
|  | OR (95% CI) | OR (95% CI) |
| AIP≤0.15 and non-HUA | Reference | |
| AIP>0.15 and non-HUA | 4.036 (3.087-5.276) ^*^ | 3.900 (2.966-5.128) ^*^ |
| AIP≤0.15 and HUA | 1.013 (0.750-1.368) | 0.960 (0.708-1.302) |
| AIP>0.15 and HUA | 6.218 (4.899-7.893) ^*^ | 5.882 (4.581-7.552) ^*^ |

Adjusted for male gender, Previous CABG, Left main disease, ACS, Diabetes mellitus, TC, LDL-C, eGFR, age, smoking, and hypertension.

*P<0.001

AIP atherogenic index of plasma, HUA hyperuricemia, OR odds ratio, and CI confidence interval.

## Table S4. Synergistic relation of AIP with HUA.

|  | Value | lower | Upper |
| --- | --- | --- | --- |
| RERI | 2.167 | 1.173 | 3.162 |
| AP | 0.349 | 0.213 | 0.485 |
| SI | 1.711 | 1.294 | 2.263 |

RERI relative excess risk due to interaction, AP attributable proportion, and SI synergy index.

## Table S5. C-statistic NRI and IDI for prediction significance of diverse models.

|  | C-statistic (95% CI) | Continuous NRI (95% CI) | IDI (95% CI) |
| --- | --- | --- | --- |
| Model 3 | 0.662 (0.643-0.682) ^*^ | Reference | Reference |
| Model 3+AIP | 0.680 (0.661-0.699) ^*^ | 0.354 (0.283-0.424) ^*^ | 0.009 (0.006-0.01) ^*^ |
| Model 3+HUA | 0.670 (0.651-0.689) ^*^ | 0.237 (0.169-0.304) ^*^ | 0.005 (0.003-0.007) ^*^ |
| Model 3+AIP+HUA | 0.752 (0.736-0.769) ^*^ | 0.750 (0.690-0.811) ^*^ | 0.073 (0.066-0.080) ^*^ |

*P<0.001

NRI net reclassification improvement, IDI integrated discrimination improvement, AIP atherogenic index of plasma, HUA hyperuricemia, and CI confidence interval.
