# Supplementary figures and images for: The synergistic effect of the atherogenic index of plasma and hyperuricemia on the prediction of coronary chronic total occlusion lesion: an observational cross-sectional study
Source: Front Cardiovasc Med. 2024 Jul 23;11:1437096. doi: 10.3389/fcvm.2024.1437096 (PMC11300285; doi:10.3389/fcvm.2024.1437096)

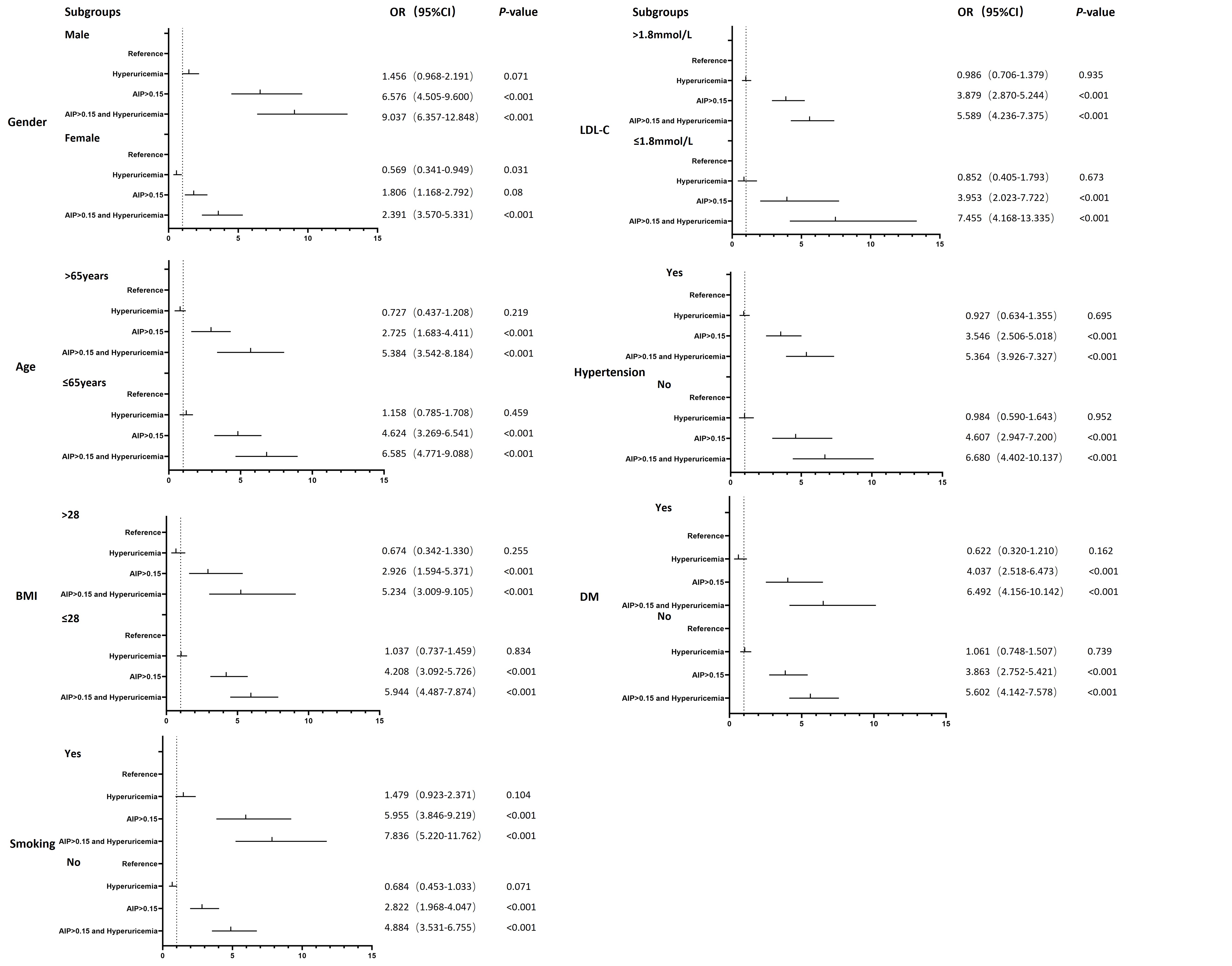

Supplement: Supplementary Figure S1 — Subgroup analysis. [file Image1.jpg]
